# Localization and dimer stability of a newly identified microbial rhodopsin from a polar, non-motile green algae

**DOI:** 10.1186/s13104-018-3181-4

**Published:** 2018-01-24

**Authors:** Peeyush Ranjan, Suneel Kateriya

**Affiliations:** 1 Department of Biochemistry, University of Delhi South Campus, Benito Juarez Road, New Delhi, 110021 India; 2 Present Address: Department of Cell Biology and Molecular Genetics, University of Maryland, College Park, MD 20742 USA; 3 Present Address: School of Biotechnology, Jawaharlal Nehru University, New Delhi, 110067 India

**Keywords:** *Coccomyxa subellipsoidea*, Proton-pumping rhodopsin, Optogenetics, Localization of the microbial rhodopsin in eukaryotes, *Chlorella vulgaris*

## Abstract

**Objective:**

The eukaryotic plasma membrane localized light-gated proton-pumping rhodopsins possesses great optogenetic applications for repolarization (silencing) of the neuronal activity simply by light illumination. Very few plasma membrane localized proton-pumping rhodopsins of a eukaryotic origin are known that have optogenetic potential. Our objective was to identify and characterize microbial rhodopsin of an eukaryotic origin that expresses on plasma membrane. The plasma membrane localized light-gated proton pump of an eukaryotic origin hold great promise to be used as an optogenetic tools for the neurobiology.

**Results:**

Here, we had characterized the cellular expression and membrane localization of a new rhodopsin in Antarctican algae *Coccomyxa subellipsoidea*. It is the first algal ion pumping rhodopsin that localizes to the plasma membrane of the eukaryotic cells. *Coccomyxa subellipsoidea* rhodopsin exists in the monomeric and dimeric state both the in vivo and in vitro. The dimeric form of the *Coccomyxa subellipsoidea* rhodopsin is resistant to heat and detergent denaturants.

**Electronic supplementary material:**

The online version of this article (10.1186/s13104-018-3181-4) contains supplementary material, which is available to authorized users.

## Introduction

Microbial type rhodopsins are sub-grouped as light sensitive proton pumps, such as bacteriorhodopsin (BR) [1], chloride pump as halorhodopsin (HR) [2] light sensitive signal transducers as the sensory rhodopsins SRI, SRII and ASR [3, 4], and light-gated ion channel, the Channelrhodopsins (ChRs) [5, 6]. Light-gated ion channels like Channelrhodopsins that are involved in phototaxis of *Chlamydomonas* [7] also excite neurons by light when heterologously expressed in it and hence used as an optogenetic tools in the neurobiology extensively. Light-gated proton pumps generated electrochemical gradient across the membrane that leads to ATP synthesis [8]. Proton pumping rhodopsins were also found in *Leptosphaeria maculans*, the benthic alga *Acetabularia* [9], and in marine eukaryotes *Oxyrrhis marina* [10]. The molecular function of an *Acetabularia* origin rhodopsin has been investigated by applying electrophysiology and spectroscopy, although their physiological roles and in vivo localization remained unclear [9–11]. Similarly, a sodium pump that is a long searched tool for neurobiology, has also been identified from a marine flavobacteria *K. eikastus* [12], but with limited success in optogenetics.

Recently, we had identified a rhodopsin sequence, CsR (*Coccomyxa subellipsoidea* rhodopsin) from the genome database of an unicellular green alga *Coccomyxa subellipsoidea* C-169 [13]. CsR generated photocurrent in oocyte was significantly higher than that of other canonical proton pumping opsins and paved the way for identification of core conserved residues responsible for maintaining directionality of pump activity [14]. In this report, we had investigated the cellular expression, location and oligomeric characterization of this proton-pumping rhodopsin in *C. subellipsoidea. C. subellipsoidea* was originally isolated from Antarctica region from extremely harsh climate, low temperature, high winds and alternating long day and night [15]. The importance of microbial rhodopsin identification from extremophiles and other drastic environmental conditions like deep seabed has been discussed in detail earlier [13]. The organisms living in harsh condition have to face low oxygen tension in surroundings. This makes difficult to the organism to maintain their cellular ATP requirement. To generate ATP, they require high membrane potential and hence proton-pumping rhodopsin with high quantum efficiency and robust plasma membrane expression can help in survival. These two features are also desirable properties of the rhodopsin to be used as an optogenetic tools. In brief, microbial rhodopsin isolated from the extremely harsh environmental condition may have better quantum efficiency and cellular expression in eukaryotes [13]. The plasma membrane expression and localization of the CsR provides a potential clue for the viability of an eukaryotic proton pumping microbial rhodopsins as a neural silencing optogenetic tool [13, 14].

## Main text

### Materials and methods

#### Identification, bioinformatic analysis of CsR, and *C. subellipsoidea* sub culturing

Search on JGI genome portal (http://genome.jgi.doe.gov) of *C. subellipsoidea* for microbial rhodopsin retrieved the sequence of CsR. Alignment was performed on ClustalW [16] platform using Bioedit. Transmembrane helices of the CsR was predicted on web based DAS transmembrane prediction tool and confirmed with other web-based tools [17]. Localization of the CsR was predicted with Plant-mPLoc [18]. Evolutionary analysis of the rhodopsins was performed using protein sequences. Sequence alignment of the rhodopsin domains was done with Clustal X 2.0. The alignment file was subjected to phylogenetic analysis by Neighbour-joining (NJ) method on MEGA 5.0 with 1000-bootstrap value [19]. Generated phylogenetic tree was also verified by maximum likelihood ML method on MEGA5 and topology was viewed by tree view [20].

*Coccomyxa subellipsoidea* C-169 culture was obtained from NIES, Japan. Related strains of *Chlorella vulgaris* (C-11 and C-12b) were procured from SAG culture collection, Germany. Cells were grown in Kuhl media under a continuous cool white light of intensity 33 µmol m^−2^s^−1^ and temperature (22 °C).

#### Heterologous expression antibody generation, immunoblotting and immunolocalization of the CsR

Human codon-adapted CsR gene synthesized by GeneArt, Germany for expression in mammalian cell-lines [14], was cloned into EcoRI and XhoI sites of pET21a expression vector using forward primer 5′-GCTGAATTCATGGCTGTGCACCAGATTGG-3′ and reverse primer 5′-TTGCTCGAGCACTTCAGCAGCTGTAGCTGG- to construct CsR-pET21a. All constructs were confirmed by automated DNA sequencing and standard protocol was adapted for expression of the CsR in BL-21 strain of *E. coli* (Additional file 1) [21–23].

Antibody was generated against a peptide (KALVSNPDGN) of the CsR. The immunoinformatics of the selected region was performed on IEDB resource, USA (http://www.iedb.org) [24]. Synthesized CsR-peptide was KLH conjugated and injected into the rabbit for raising polyclonal antibody using Merck, India. Total cell lysate (TCL) of* C. subellipsoidea*, * Chlorella vulgaris* C-11b and C12 were prepared as described elsewhere [21]. Cells in early exponential growth phase were harvested by centrifugation and cell pellets were re-suspended in PBS (PBS; 150 mM NaCl, 10 mM sodium phosphate, and pH 7.2) in the presence of protease inhibitor cocktail (Sigma, USA). Cells were lysed by sonication (8 s on and off pulse for 8–10 times). Samples were solubilized by mixing cell lysate for different time and temperature as mentioned in the result section and in figure legend. TCL was separated on 12% SDS-PAGE and transferred onto nitrocellulose membrane for immunoprobing. Protein blotted membrane was blocked with 5% non-fat dry milk powder in PBST (PBS + 0.1% tween-20; Sigma USA) for 1 h at room temperature. Blocked membrane was incubated with CsR antibody (1:3000) dilutions. Immunolabelling was done using horseradish peroxidase conjugated anti-rabbit secondary antibody (Sigma, USA 1:5000) and visualized by the standard ECL method.

Immunolocalization of the CsR in *C. subellipsoidea* was performed as described earlier [21, 23]. A 150 µl of cell suspension was seeded on poly-l-Lysine (Sigma, USA) coated coverslip and fixed with 4% paraformaldehyde (Sigma, USA) in PBS. Cells on the coverslips were permeabilized with chilled 100% ethanol for 10 min at − 20 °C. Permeablized cells were washed with PBS + 0.25 M NaCl for 10 min, followed by PBS for 5 min at room temperature. After brief washings with PBS containing 0.5% triton X-100 (PBST), cells were incubated with freshly diluted anti-CsR antiserum (1:1500) or KLH antiserum or pre-immune serum for overnight at 4 °C. After 3X washing with PBST, samples were incubated with Fluorescein Iso-thiocynate conjugated (FITC) secondary antibody (Sigma, USA. 1:2500) for 1 h at room temperature. Followed by brief washings, anti-fade reagent (slow fade gold, molecular probes) was applied on coverslips before mounting of the slides. Plasma membrane was labeled by tracker dye FM 4-64Fx (Invitrogen, USA) at concentration 5 µg/ml [23]. Images were captured with Leica TCS SP5 confocal microscope at central instrument facility, UDSC, New Delhi, India.

### Results

#### CsR is closely related to algal light-gated proton pump

**Fig. 1:**
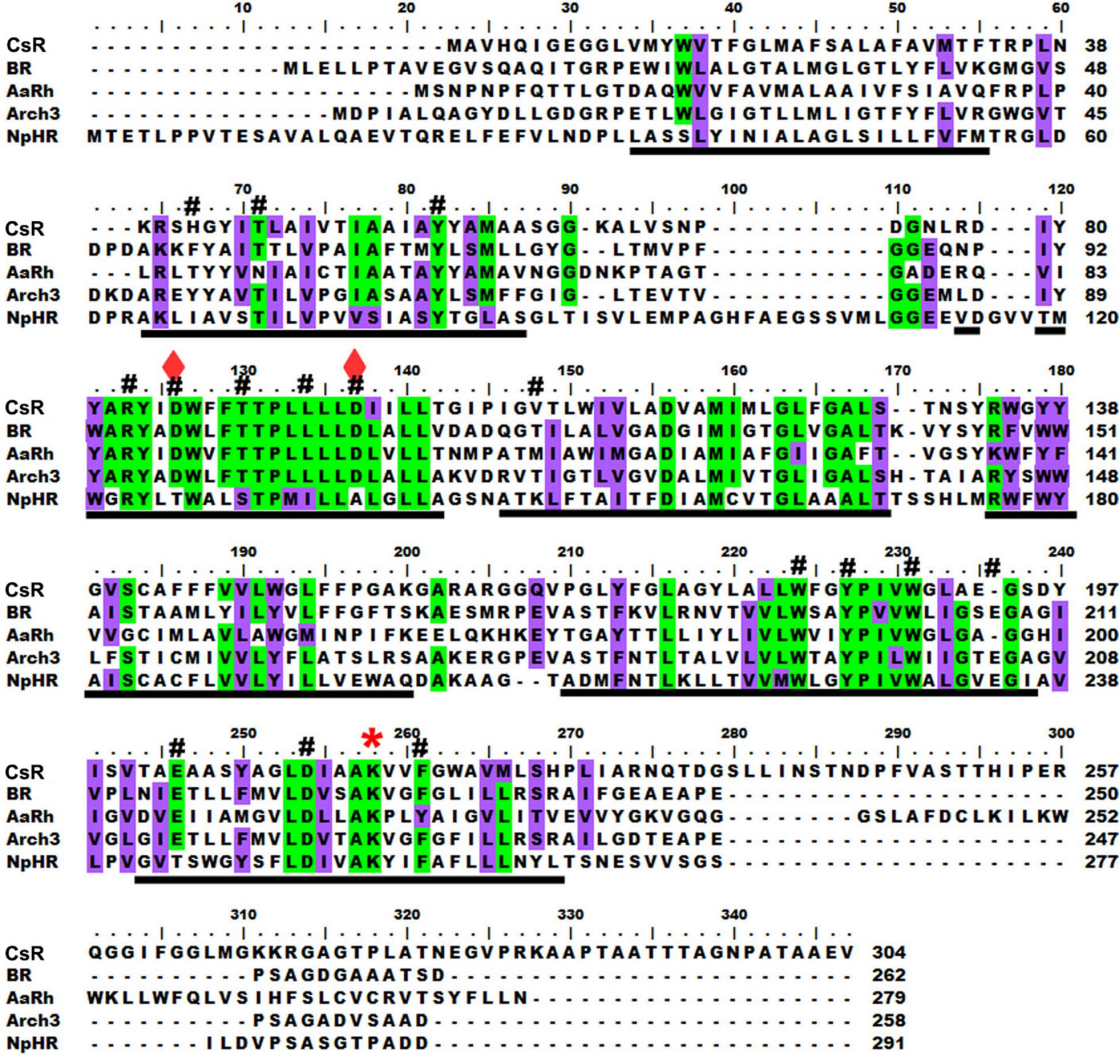
Comparative sequence analysis of the CsR with other proton-pumping rhodopsins. The green and purple background depicts the identical and similar residues, respectively. Black bar below alignment depicts the predicted transmembrane regions. The red star depicts the conserved lysine residue and #depicts the conserved proton transport pathway of the CsR

Database search of the *C. subellipsoidea* on JGI (V 2.0) and phytozome portal for the rhodopsins revealed the presence of a new microbial type rhodopsin. Identified CsR rhodopsin showed homology with bacteriorhodopsin and *Acetabularia* rhodopsin [9]. Transmembrane helices were analyzed by transmembrane prediction from different web-tools. More than 40% homology was observed for CsR with that of the known bacteriorhodopsin and *Acetabularia* rhodopsin. Retinal binding lysine motif (red star marked in Fig. 1, adapted from [14] was found to be conserved in the seventh helix. The residues involved in proton transport pathway of the BR were also conserved in CsR (#, Fig. 1). Aspartic acid residues, which act as proton acceptor and donor respectively, were well conserved (quadrilateral) in CsR, which suggested that CsR is a putative light-gated proton pump. An m-locP [18] predicted CsR localization on the plasma membrane (data not shown).

#### CsR stayed in monomeric and dimeric form in vivo and in vitro

**Fig. 2:**
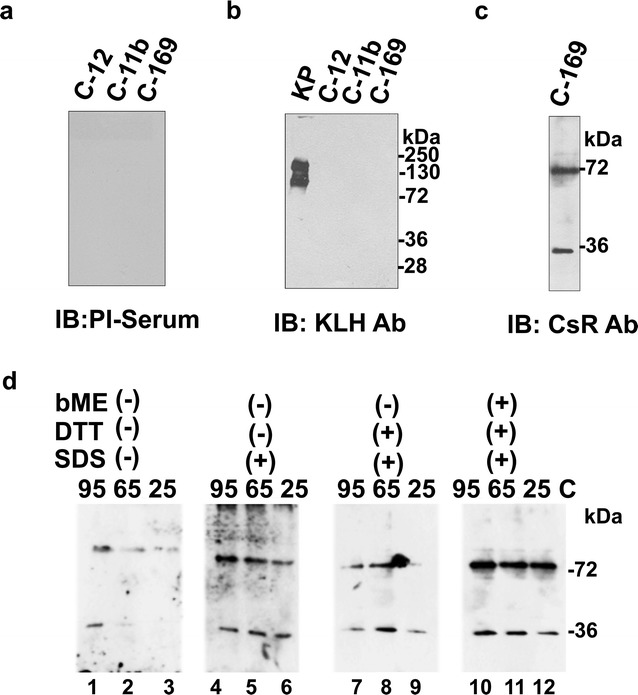
Presence of the CsR protein and cellular expression in *C. subellipsoidae* C-169. The TCL of *C. subellipsoidea* and two other closely related *Chlorella vulgaris* strain were probed with **a** pre-immune serum (Dilution; 1:3500), **b** KLH antibody (Dilution; 1:6000), with KLH protein loaded with TCLs, **c** western blot profile of TCL of C-169 probed with CsR antibody (Dilution; 1:3500). **d** Dimer profiling of CsR in *C. ellipsoidea*. The sample prepared in three denaturing reagents (SDS, DTT, βME) and incubated at three different temperatures and probed with anti CsRAb without denaturant (lane 1–3), with SDS (lane 4–6), with SDS and DTT (lane 7–9) and with SDS, DTT and βME (lane 10–12). The immunoblots are representative image of three different experimental sets

Epitope prediction, antigenicity and surface accessibility of the CsR protein was performed using IEDB (http://www.iedb.org), the ten amino acid long epitope (KALVSNPDGN) was selected for raising antibody. Pre-immune serum, anti-KLH Ab (Abcam, UK) and Penta His Ab (Qiagen, UK) was used as control. The cross reactivity of Pre-immune serum was checked with TCL of C-169, C-11b, and C-12. On western blotting, no protein band was detected in TCL of C-169, C-11b, and C-12 by pre-immune serum (Fig. 2a). The TCL of C-169, C-11b, and C-12 along with KLH protein (Pierce, USA) was also probed with KLH antibody, a strong band of KLH protein was observed with KLH protein and no protein band was detected in TCLs (Fig. 2b). Anti-CsRAb and anti-Penta-His Ab detected the similar band pattern with recombinant CsR (Additional file 1). The anti-CsR antibody detected two protein bands of apparent monomer and dimer of CsR (35 and ∼70 kDa) in TCL of *C. subellipsoidea* (Fig. 2c).

The role of temperature and denaturant on monomeric and dimeric state of CsR was studied at different temperatures (25, 65 and 95 °C) without any detergent (SDS) and with DTT and bME. Under non-denaturing and non-reducing condition (i.e., without SDS, DTT, and bME) and at temperature (25 and 65 °C), CsR was present in dimeric form (Fig. 2d; Lane 1, 2). The monomer and dimer were  observed in sample prepared at 95 °C non-denaturing conditions. The dimer and monomer bands were also prominent in all other conditions (Fig. 2d; lane 3–12). CsR mostly stayed in dimeric form that was mostly stable in denaturing conditions and no complete loss of dimer was observed. Dimer was prominent with recombinant protein sample and not enough protein was produced to purify in the native form (Additional file 1). Presented results suggested that dimer of CsR was present both in TCL of C-169 and bacterial total lysate expressing CsR.

#### CsR localizes onto plasma membrane

**Fig. 3:**
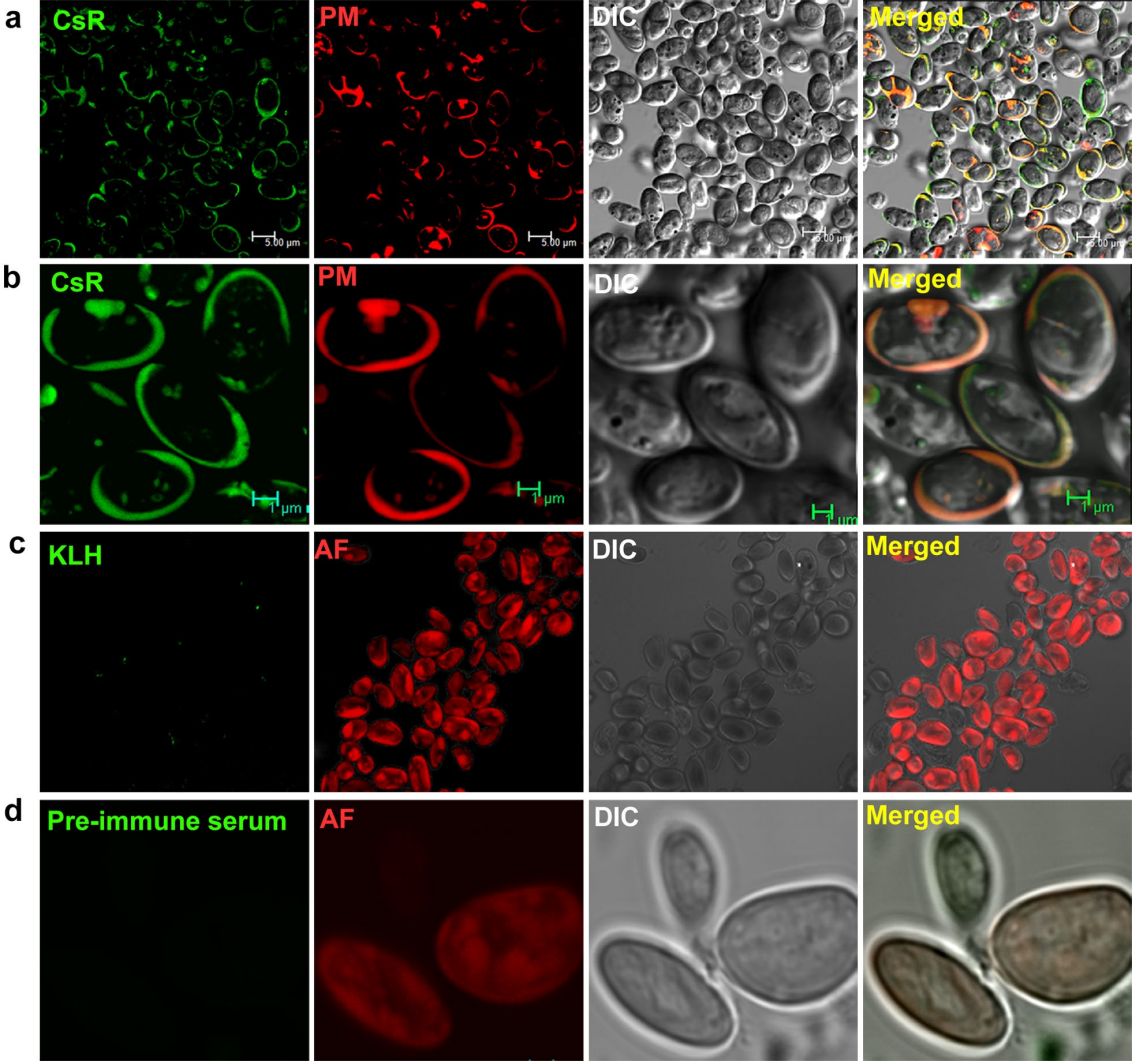
Plasma membrane localization of the CsR in *C. subellipsoidea* cells. **a** Cells probed with anti-CsR shows plasma membrane localization in the green channel. Plasma membrane (PM) specific FM4-64Fx dye is shown in the red channel. Cells are shown in DIC image. **b** Magnified view of the few cells from in the microcopy same field, to show detailed localization pattern of the CsR. **c** No signal was observed when these cells were probed with KLH antibody. Cells were marked by autofluorescence (AF) and DIC. **d** No signal was observed with pre-immune serum as well. Cells are shown by autofluorescence (AF) and DIC

Immunolocalization of the CsR using anti-CsR antibody confirmed the plasma membrane localization of the CsR (Fig. 3). CsR signal was present on the periphery of the cells and co-localized with plasma membrane tracker red dye FM4-64Fx. No signal was observed when cells were probed with the anti-KLH antibody and pre-immune serum (Fig. 3), respectively.

## Discussion

CsR is the first light-gated proton pump found in the fresh water, unicellular, non-flagellate, green algae. The retinal chromophore-binding signature was conserved in CsR (Fig. 1). The genome database mining for enzymes involved in retinal metabolism pathway were also present in the genome database of *C. ellipsoidea* (Beta, beta-carotene 15,15-dioxygenase, retinol dehydrogenase), which supports existence of the retinal binding protein in this organism. Archaeal rhodopsin sequences are known in archaea, eubacteria and lower eukaryotes [25]. Two light-gated proton pumps were characterized from marine alga *Acetabularia*. This report describes the characterization of light-driven proton pumping rhodopsin from a unicellular, non-motile freshwater alga. Phyletic relatedness analysis showed that BR and proteorhodopsin were the next closest rhodopsins to CsR (Additional File 2; highlighted in blue and pink). CsR stayed in dimer form both in vivo and in vitro (Fig. 2). The CsR was localized on the plasma membrane (Fig. 2). Plasma membrane localization may help in the maintenance of the membrane voltage under certain conditions. It would be interesting to know the physiological significance of the oligomer of the CsR and its localization on plasma membrane of the green algal cells.

The light-gated ion pumps are used to hyperpolarize the membrane of the electrically excitable cells that leads to shut down the depolarization evoked by complimentary light-gated channels like Channelrhodopsin leading to precise on and off control of cellular system through optogenetics [26, 27]. Few rhodopsins (halorhodopsin or archaerhodopsin-3) are capable of silencing the neural excitabilities by a hyper-polarization of the membrane upon illumination [28]. The membrane targeting of the Ar-3 or HR is poor due to lower expression, poor trafficking of a prokaryotic protein in the eukaryotic system or lack of signal target sequence [13]. The expression level and localization are the important parameters for ion pumping rhodopsin due to their fixed stoichiometric of one charge transferred per absorbed photon. The strong signal peptide sequence of 28 amino acids was predicted for the CsR that can be tested for the effective membrane localization of the CsR and other archaeal rhodopsins as well in the target mammalian system. Localization of the CsR in the plasma membrane of *Coccomyxa* suggests that it might be a useful optogenetic tools for the silencing of the excitable cells. Moreover, mutational analysis of the CsR helped in the elucidation of of basic mechanism and role of amino acids involved in the proton pumping activity. CsR also elucidated the amino acid residues involved in conversion of pump to Channelrhodopsin [14].

## Limitations

Poor expression and plasma membrane localization of an archaeal rhodopsin in mammalian system poses great challenge for optogenetic tools. Further, investigations are required to characterize the link of dimerization of CsR with its light-gated ion conductivity or signaling state.

## Additional files

**Additional file 1.** Recombinant CsR expressed in *E. coli* were probed with anti-Penta His Ab and anti-CsR Ab.

**Additional file 2.** Phylogenetic relatedness of CsR with rhodopsins from different taxa of life.

## Abbreviations

ASR: anabaena sensory rhodopsin
BR: bacteriorhodopsin
ChRs: Channelrhodopsins
CsR: *Coccomyxa subellipsoidea* rhodopsin
FITC: Fluorescein Iso-thiocynate
HR: halorhodopsin
KLH: keyhole limpet hemocyanin
ML: maximum likelihood
NJ: neighbour joining
PBS: phosphate buffer saline
SRI & SRII: sensory rhodopsin I & II
TCL: total cell lysate
