# Supplementary material for: Localization and dimer stability of a newly identified microbial rhodopsin from a polar, non-motile green algae
Source: BMC Res Notes. 2018 Jan 24;11:65. doi: 10.1186/s13104-018-3181-4 (PMC5781313; doi:10.1186/s13104-018-3181-4)
Supplement: Supplementary file 1 — Additional file 1. Recombinant CsR expressed in E. coli were probed with anti-Penta His Ab and anti-CsR Ab. [file 13104_2018_3181_MOESM1_ESM.pdf]

Additional File 1

Localization and Dimer Stability of a Newly Identified Microbial Rhodopsin from a Polar, Non-motile Green Algae

Peeyush Ranjan[1, 2] and Suneel Kateriya[1, 3*]

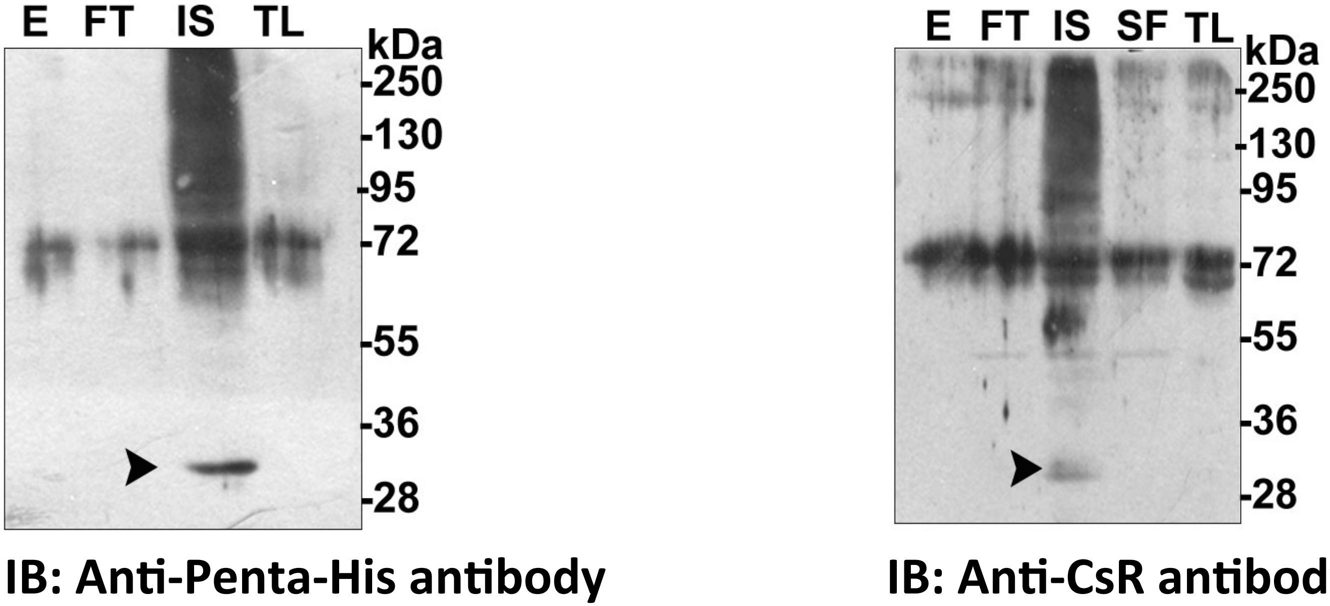

BL21 strain containing CsR-pET21A were grown in TB media at 37 °C. Induction was done at O.D600 0.6-0.7 with 1 µM IPTG and incubated with 10 µM all-trans retinal for overnight in dark. Cells were harvested at 4000 rpm, resuspended in 1XPBS and sonicated. Cell lysate was centrifuged at 13000 rpm for 1hr at 4 °C. Pellet was solublized in 1X PBS with 1.5% DM for 8 hrs. The solubilised fraction was spin down at 100,000 g for 1hr at 4 °C. Supernatant was incubated with cobalt bead for binding. Washing was done with 0.15% DM in PBS and protein was eluted with 0.05% DM with 300 mM imidazole in PBS.

To check the specificity of CsR Ab, expression profile of CsR in *E.coli* was probed with both Anti-CsR Ab and Anti-Penta His Ab. Almost similar profile was observed in both case. Majority of the protein was present in insoluble fraction and very less enrichment in elution, as expected for seven membrane proteins, was observed. **TL: Total lysate of *E. coli*, IS: Insoluble fraction, SF: Soluble fraction, FT: Flow through, E: Elution.**
