# Supplementary material for: Localization and dimer stability of a newly identified microbial rhodopsin from a polar, non-motile green algae
Source: BMC Res Notes. 2018 Jan 24;11:65. doi: 10.1186/s13104-018-3181-4 (PMC5781313; doi:10.1186/s13104-018-3181-4)
Supplement: Supplementary file 2 — Additional file 2. Phylogenetic relatedness of CsR with rhodopsins from different taxa of life. [file 13104_2018_3181_MOESM2_ESM.pdf]

Localization and Dimer Stability of a Newly Identified Microbial Rhodopsin from a Polar, Non-motile Green Algae

Peeyush Ranjan1, 2 and Suneel Kateriya1, 3*

Microbial rhodopsins from different taxa were selected for phyletic relatedness analysis. Closest relatives of CsR were light-activated proton-pumping rhodopsins from *Acetabularia* (AaRhI & II). CsR grouped with algal rhodopsin of AaRh forming a separate clade and were highlighted in the green. BR and proteorhodopsin were the next closest sequence to CsR (Highlighted in blue and pink). Sensory rhodopsins (highlighted in orange) and channelrhodopsins were distantly related to the CsR (red).

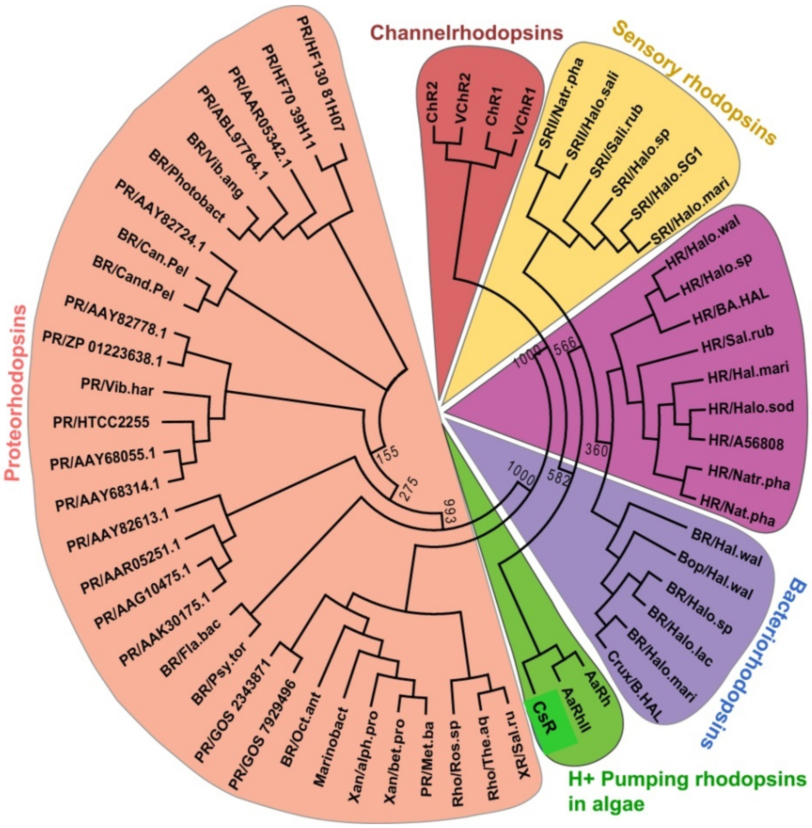

Evolutionary analysis of CsR protein sequence with diverse microbial rhodopsins protein. CsR groups with *Acetabularia* rhodopsin sequences and highlighted with green. Sensory rhodopsins were separated as shown by yellow boxes. Chloride ion pumping halorhodopsins are highlighted by purple background. Proteorhodopsins are highlighted by pink background and the blue background highlights bacteriorhodopsins. Numbers represent bootstrap value.
